# A case of Metaplastic atrophic gastritis in immune Dysregulation, Polyendocrinopathy, Enteropathy, X-linked (IPEX) syndrome

**DOI:** 10.1186/s12887-018-1169-9

**Published:** 2018-06-15

**Authors:** Youyou Luo, Jie Chen, Youhong Fang, Jingan Lou, Jindan Yu

**Affiliations:** Gastroenterology Department, Children’s Hospital, Zhejiang University School of Medicine, Hangzhou, China

**Keywords:** Gastric atrophic metaplasia, Immune dysregulation, Polyendocrinopathy, Enteropathy, X-linked (IPEX) syndrome, Forkhead box protein 3, Ciliated cell

## Abstract

**Background:**

Autoimmune metaplastic atrophic gastritis is a chronic progressive inflammatory condition. The clinical spectrum includes pernicious anemia, atrophic gastritis, antibodies to parietal cell antigens and intrinsic factor, achlorhydria, hypergastrinemia and carcinoma. It is rare in paediatric cohorts.

**Case presentation:**

We present the case of a boy with metaplastic atrophic gastritis in whom immune dysregulation, polyendocrinopathy, enteropathy, X-linked(IPEX) syndrome was confirmed by *FOXP3* gene mutation. The patient was referred to the hospital at the age of 3 years with recurrent emesis, diarrhoea and malnutrition. His elder brother died at 9 years of age from acute respiratory distress syndrome and renal tubular acidosis. The patient was allergic to cow milk formula and noodles. Oesophagegastroduodenoscopy revealed redness, erosion and edema throughout the stomach; whitish granules in the duodenal bulb; and edema in the second part of the duodenum. Biopsies showed extensive villous atrophy and goblet cell depletion in the duodenum. He was diagnosed with type-1 diabetes mellitus (T1DM) during the treatment of methylprednisolone. Serum antibodies against glutamic acid decarboxylase and pancreatic islets were detected. The patient’s FOXP3 gene was sequenced; this identified that the patient was hemizygous for a pathogenic variant [NM_014009.3:c.748_750del (p.Lys250del)].

**Conclusion:**

Metaplastic atrophic gastritis is rarely reported in patients with IPEX. Clinical gastroenterologists should be aware of IPEX syndrome when facing the complex syndromes of metaplastic atrophic gastritis and endocrinopathy.

## Background

Immune dysregulation, polyendocrinopathy, enteropathy, X-linked (IPEX) syndrome is a rare disorder caused by mutations in the forkhead box protein 3 (*FOXP3*) gene, which is a master transcriptional regulator for the development and function of CD4+ regulatory T (Treg) cells [1]. *FOXP3*+ Treg cells are essential for immune homeostasis and tolerance to self and nonself antigens [2]. The dysfunction of these cells leads to the multi-organ autoimmunity that characterizes IPEX syndrome.

Metaplastic atrophic gastritis is defined as the loss of gastric oxyntic and/or antral glands, metaplastic epithelial changes, and increased lamina propria inflammation [3]. It can be associated with long-standing *H. pylori* infection and with autoimmune causes. *H. pylori*-induced gastritis may progress to autoimmune metaplastic atrophic gastritis. It is thought that the aetiology of this progress is antigenic mimicry or cross-reactivity. Atrophic gastritis is classically a disorder of adults. Whereas, in children, it is rare even in association with *H. pylori* infection [4]. Two typical types of metaplasia that occur in atrophic gastritis include pseudopyloric metplasia and intestinal metaplasia. In addition, pancreatic, ciliated cell, and squamous metaplasia are rare in paediatric cohorts [5, 6]. Here we present the case of a pediatric patient with metaplastic atrophic gastritis in whom IPEX syndrome was confirmed by *FOXP3* gene mutation.

## Case presentation

The patient was referred to the hospital at the age of 3 years and 11 months. He was born at term with a birth weight of 3400 g after an uneventful pregnancy. The parents were nonconsanguineous and healthy. The mother had been pregnant twice. The first son died at 9 years of age from acute respiratory distress syndrome and renal tubular acidosis. The patient was exclusively breastfed and had no problem during the first 7 months of life. However, he developed severe vomiting and watery diarrhoea after the ingestion of noodles. Similar symptoms were observed after he drank cow’s milk. He continued exclusive breast-feeding until he was 19 months old. Then, soy bean milk and rice soup were introduced, which were well tolerated. Growth was normal up to 3 years of life. At the age of 3 years, whole protein formula was introduced in his diet. Three months later, he presented vomiting, watery diarrhoea, hepatitis, hypoalbuminemia and anaemia. His body weight for age rapidly decreased below the 3rd percentile in half year. The parents stopped whole protein formula and reintroduced amino acid formula in his diet. He was hospitalized on several occasions in a local hospital because of dehydration and severe malnourishment.

**Fig. 1:**
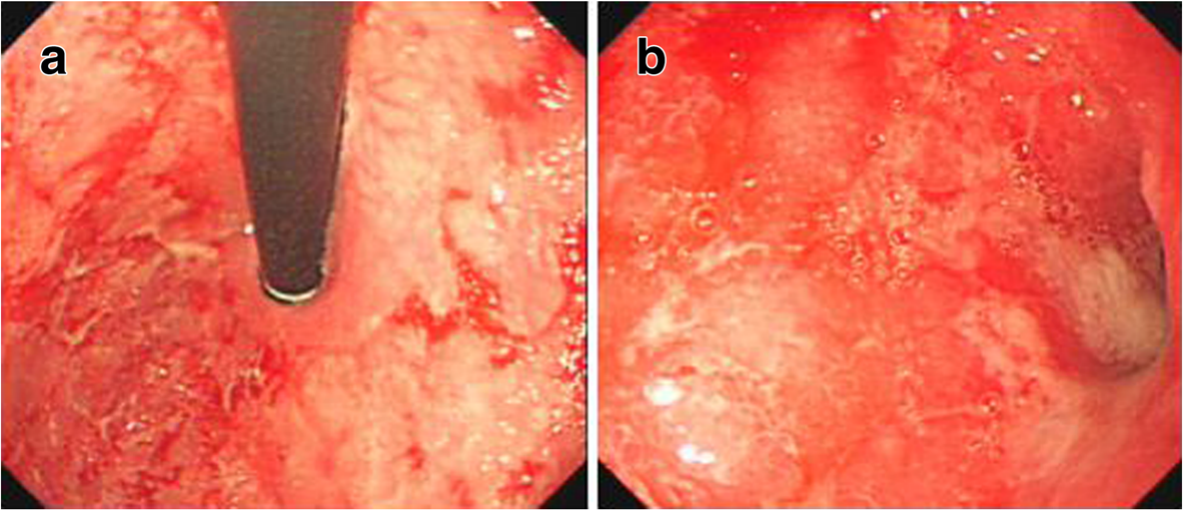
Endoscopic manifestations of the patient. (**a.** Redness, erosion and edema in the antrum. **b.** Redness, erosion and edema in gastric fundus)

On admission, his weight was 9.2 kg (below the 3rd percentile for age), and his height was 99 cm (17th percentile for age). Anaemia (haemoglobin 78 g/L; reference range 110–155 g/L) and hypoalbuminemia (albumin 31.6 g/L; reference range 32–52 g/L) were detected. Liver function showed elevated glutamic-pyruvic transaminase levels (111 U/L; reference range < 50 U/L). Immunoglobin G (IgG) anti-endomysium antibody and IgG anti-gliadin antibody were positive. Amino acid and acyl carnitine analyses for metabolic disorders were negative. He had a slightly increased serum immunoglobin E (IgE) level (221 IU/ml; reference range 0–100 IU/ml), while the concentrations of serum IgG, immunoglobin A (IgA) and IgM were within normal ranges. Oesophagegastroduodenoscopy (EGD) demonstrated redness, erosion and edema throughout the stomach (Fig. 1). Whitish granules could be seen in the duodenal bulb and edema in the second part of the duodenum. Villi showed extensive atrophy and goblet cell depletion in the duodenum. Significant eosinophil infiltration (15–20 eos/HPF) was found in biopsies of the stomach and duodenal bulb. Immunohistochemistry for *H. pylori* was negative. Colonoscopy showed edema in the ascending, transverse and descending parts of the colon, but no active inflammation was seen and no prominence of eosinophils was identified.

The boy was diagnosed with metaplastic atrophic pangastritis. He was treated with methylprednisolone and total parenteral nutrition. On the second day after the treatment, he experienced hyperglycaemia (blood glucose level up to 20.2 mmol/L; reference range 3.3–7.8 mmol/L). His serum antibodies against glutamic acid decarboxylase and pancreatic islets were positive. He was diagnosed with type-1 diabetes mellitus (T1DM), and insulin was initiated. Clinical response was achieved after he received two-month methotrexate (10 mg/m^2^.w). Steroids and insulin were gradually tapered over 6 months. The boy changed to exclusive enteral nutrition with amino acid formula. During his hospitalization, he suffered from severe pneumonia and catheter-associated infection. Repeat EGD showed a similar appearance. Biopsies from the stomach and duodenal bulb indicated the absence of metaplasia but similar eosinophil infiltration (10–30 eos/HPF). We introduced mercaptopurine (1.3 mg/kg.d), which kept him in clinical remission for 1 year.

Now he is 5 years and 9 months of age. His weight for age reaches the 5th percentile. His height for age is the 2nd percentile. The diagnosis of IPEX was confirmed by molecular analysis using EDTA whole blood, which revealed a pathogenic variant in the leucine zipper of *FOXP3* [NM_014009.3:c.748_750del (p. Lys250del)] which is associated with IPEX. The mother was subsequently tested and identified as a heterozygous carrier for this variant.

## Discussion and conclusions

The distinctive symptoms of IPEX syndrome present as early onset of intractable diarrhoea, T1DM, and eczema [7]. The onset of the disease usually occurs in males within the first months of life and can be fatal if not well treated. However, IPEX syndrome has a wide spectrum of clinically manifested autoimmune diseases, such as thyroiditis, haemolytic anaemia, thrombocytopenia, neutropenia, renal disease, arthritis, and hepatitis [5]. Many patients begin early with a milder form of the disease, which is often misdiagnosed or delayed in diagnosis. The present case we present here is not a classical presentation of IPEX. He presented later than typical IPEX patients. Furthermore, his primary manifestation was metaplastic atrophic pangastritis, which has rarely been reported.

In previous reports, there were three patients with IPEX syndrome who had a *FOXP3* mutation identical to that of our patient [8–10]. However, the phenotype of each case was different (Table 1). This might indicate the potential role of environmental and epigenetic factors in determining the features of the disease.

**Table 1:** Clinical features and outcomes in IPEX patients with the same deletion (c. 748–750 del AAG) in *FOXP3* gene

References	Age at onset	Age at diagnosis	Diarrhoea	T1DM	Eczema	Additional Clinical findings	IgE (IU/ml)	Family history	Therapy	Outcome
8	2 months	9 years	+	+	–	Arthritis, ITP, mild hepatitis	na	A brother died at 15 months of age with similar clinical presentation.	CS, CSA, tacrolimus, rofecoxib, MTX, infliximab, rituximab, BMT	Died from respiratory distress on post-BMT day 94.
9	2 months	5 years	vomiting	+	+	MCNS, food allergy, haemolytic anaemia, sepsis	1141	Mother’s two brothers died at the age of 1 year due to sepsis.	CSA, CS	na
10	2 months	4 years	+ Celiac disease-like pattern in small bowel biopsies	+	+	AIH, anaemia	N	–	CS, CSA, AZA, BMT	Alive, 6 years

*na*, not available; N, within normal ranges; *ITP*, idiopathic thrombocytopenic purpura; *MCNS*, minimal change nephrotic syndrome; *AIH*, autoimmune hepatitis; *CS*, corticosteroid; *CSA*, cyclosporine; *MTX*, methotrexate; *MP*, mercaptopurine; *AZA*, azathioprine; *BMT*, Bone marrow transplantation

Metaplastic atrophic gastritis and duodenal atrophy are atypical manifestations in IPEX syndrome. To our knowledge, gastritis has rarely been reported in patients with IPEX [6, 11, 12]. Only one was indicated to have ciliated cells in the gastric fundus [6]. Normally, lymphocytic, plasmocytic, neutrophil and eosinophil infiltration can be found in the duodenum, stomach and colon [13]. IPEX syndrome includes multi-organ autoimmune disorders. Gastritis in IPEX might be caused by autoimmune issues. In a study of intestinal morphological changes with IPEX children [12], the histological features of gastrointestinal biopsies were divided into three types: graft-vs-host disease-like pattern, celiac disease-like changes, and enteropathy with a complete depletion of goblet cells. However, these inflammatory changes are nonspecific. In our case, according to microscopic manifestations from biopsy, the diagnosis of autoimmune atrophic gastritis was suspected.

The presentation of our patient is not classic IPEX syndrome because of late-onset symptoms and metaplastic atrophic gastritis. The mechanism of the variable phenotype of the same *FOXP3* mutation needs to be further studied.

## Abbreviations

EGD: Oesophagegastroduodenoscopy
*FOXP3*: Forkhead box protein 3
IgA: Immunoglobin A
IgE: Immunoglobin E
IgG: Immunoglobin G
IPEX: Immune dysregulation, polyendocrinopathy, enteropathy, X-linked
T1DM: Type-1 diabetes mellitus
